# Study protocol for the PHANTOM study: prehospital assessment of noninvasive tissue oximetry monitoring

**DOI:** 10.1186/s13049-014-0057-z

**Published:** 2014-10-03

**Authors:** Andrew Weatherall, Alan Garner, Nigel Lovell, Stephen Redmond, Anna Lee, Justin Skowno, Jonathan Egan

**Affiliations:** CareFlight, Westmead, NSW Australia; The Children’s Hospital at Westmead, Westmead, NSW 2145 Australia; The University of Sydney, Westmead, NSW 2145 Australia; The University of New South Wales, Randwick, NSW 2031 Australia; The Chinese University of Hong Kong, Shatin, NT Hong Kong

**Keywords:** Traumatic brain injury, Near-infrared spectroscopy, Prehospital

## Abstract

**Background:**

Traumatic brain injury is a major cause of mortality and morbidity worldwide. It can be worsened by secondary injury particularly with hypoxia or hypotension. Current prehospital guidelines emphasise regular measurement of peripheral oxygen saturation and blood pressure but there is no monitor in use to provide direct information relating to blood flow or oxygen delivery to the brain tissue. This prospective cohort study will assess the utility of near-infrared spectroscopy monitoring in prehospital medicine in demonstrating injury, pathophysiology and associations with long-term functional outcomes.

**Methods/design:**

A prospective cohort study will be conducted in prehospital services where physician/paramedic teams respond rapidly to patients suffering significant traumatic injuries. A study observer accompanying the clinical team will apply non-invasive near-infrared spectroscopy tissue oximetry using a Nonin EQUANOX 7610 Regional Oximetry monitor (TM Nonin Medical, Inc.). This will be applied to patients with traumatic injuries less than 30 minutes old requiring transport. Measurements will be taken at two sites on the forehead and one on the forearm. Clinical teams will be blinded to all monitoring values. Near-infrared spectroscopy tissue oximetry parameters of oxyhaemoglobin%, deoxyhaemoglobin%, total tissue haemoglobin index and regional oxygen saturation will be recorded. Separate statistical analysis relating to time spent with cerebral regional oxygen saturation values < 45% and time series analysis will be performed to demonstrate associations with acute phase outcomes including injuries seen on cerebral imaging, and long-term functional outcomes measured by Glasgow Outcome Score and Extended Glasgow Outcome Score will then be undertaken.

**Discussion:**

This prospective cohort study will demonstrate associations evident from the earliest stages of prehospital treatment between near-infrared spectroscopy tissue oximetry values and both acute and long-term outcomes of patients suffering traumatic injuries. This may provide the basis for future interventional studies utilising near-infrared spectroscopy tissue oximetry to guide prehospital trauma care.

**Trial registration:**

This trial is registered with the Australian and New Zealand Clinical Trials Registry. The registration number is ACTRN12611001124921.

## Background

Traumatic brain injury (TBI) is a significant cause of death and severe disability [1]. Estimates suggest that long term costs of caring for people with moderate and severe TBI add $8.6 billion to health spending per year in Australia [2]. Despite advances in hospital care of patients with TBI, minimal improvements in outcome have been evident over the last twenty years [1].

In the setting of TBI, it is not only the initial injury that is responsible for patient outcome. After the initial insult, the brain is susceptible to further damage if normal physiologic conditions are not restored. Such insults include low blood pressure or hypoxia [3,4]. The primacy of hypoxia and hypotension in the debate has guided the development of consensus guidelines for prehospital management of patients with TBI with an emphasis on regular blood pressure measurements and checking oxygen saturations [5]. However, having this knowledge to guide monitoring care has not resulted in significant changes to patient outcomes.

Peripheral measurements such as non-invasive blood pressure and pulse oximetry remain relatively crude measurements distant to the tissue of most interest; the brain itself. Additional monitoring options that have become available in the hospital such as jugular venous bulb oximetry, direct tissue oximetry and intracranial pressure measurement are not appropriate to the prehospital environment [6-8]. In the New South Wales metropolitan setting, the time spent in the prehospital treatment phase before reaching a trauma centre averages over one hour [9]. This represents a significant period of vulnerable time where monitoring information relevant directly to cerebral tissue is absent.

Near-infrared spectroscopy (NIRS) tissue oximetry was first described in 1977 and is now used within hospitals [10]. It uses light in the near-infrared spectrum (700–1300 nm) and relies on the interaction of these wavelengths with tissue chromophores to derive information about oxygenated blood in the monitored region. Commercial devices use reflectance spectroscopy. The light is shone into tissues from an emitting light source within a self-adherent probe at the skin. Light entering the tissues interacts with chromophores, with the degree of absorption and scattering specific to the near-infrared wavelength employed. The fraction of transmitted light that returns to the surface is detected within the same probe, with a standard separation between the initial light source and the detector.

Utilising known characteristics of the interaction of particular near-infrared wavelengths with biological tissues allows the calculation of a number of parameters:

- Oxyhaemoglobin%, (HbO_2_) a measure of haemoglobin carrying oxygen through the tissues;
- Deoxyhaemoglobin% (HbD), a measure of haemoglobin present but not carrying oxygen;
- Total tissue haemoglobin index (HbT) – an index (expressed in g/dL) corresponding with blood volume and flow within the sampled tissue; and,
- Regional oxygen saturation (StO_2_%), providing a measure of the balance of oxygen delivery and utilisation in the tissue under the probe as it adjusts for the influence of chromophores other than oxy- and deoxyhaemoglobin as well as the adjustment for the proportional amounts of arterial and venous blood in the sampled area.

Recent monitors display improved accuracy and time resolution and are therefore spreading into a variety of clinical areas including detection of silent ischaemia in subarachnoid haemorrhage, coronary artery bypass grafting, aortic arch surgery, paediatric cardiac surgery and carotid endarterectomy [11-15].

NIRS oximetry has been utilised in head injury patients although application of the monitor has generally occurred hours after the initial injury. Early work with NIRS tissue oximetry has suggested that it may provide useful information in detecting intracranial haematomas and correlation with direct tissue oximetry. To date this work has been entirely hospital-based [16-19].

A further relatively recent development in the use of NIRS oximetry is the ability to use novel analysis techniques to assess dynamic factors such as cerebrovascular autoregulation. Various approaches such as coherence analysis of spectral waveforms and floating correlation analysis of moving value windows have been described in the assessment of vascular reactivity in subarachnoid haemorrhage, traumatic brain injury, paediatric cardiac surgery and extracorporeal membrane oxygenation [20-24]. Examples of this analysis rely on examination of the trends and patterns seen in the haemoglobin index (HbT) rather than analysis of absolute values of either HbT or rSO_2_.

This study builds on earlier work demonstrating the possibility of obtaining NIRS oximetry monitoring values in the prehospital and transport environments [25]. It will assess the utility of prehospital NIRS oximetry in the monitoring of patients with TBI.

### Study plan

The *hypothesis* is that NIRS tissue oximetry can provide direct real-time cerebral physiological data that provides an insight into rapidly developing brain pathology in TBI and its management, as well as early associations with long-term outcomes. Three aims will be delivered to achieve this:

1. To establish methods of analysing the NIRS tissue oximetry monitoring parameter time series that correspond with evolving pathology in the TBI.
2. To establish the association between NIRS tissue oximetry monitoring parameters and acute pathology in patients with TBI.
3. To establish the association between NIRS tissue oximetry monitoring parameters and the long-term recovery of patients after TBI, as assessed at follow-up to 12 months.

This research will therefore be conducted in a prehospital setting, providing early monitoring data in patients likely to have TBI with a plan for subsequent follow-up data collection to examine both acute and long-term outcomes. Novel analysis methods as featured in Aim 1 will provide better options for demonstrating the associations described in aims 2 and 3.

### Study design and setting

This is a prospective cohort study.

This research will be conducted in a prehospital emergency medical service employing a physician-paramedic model of care to deliver rapid response trauma care and employing both rotary-wing and road transport modalities. The clinical team is capable of providing advanced life support, anaesthesia, intubation and ventilation, blood transfusion and peripheral haemorrhage control manoeuvres along with other critical care interventions at the scene of the accident or during patient transport.

### Inclusion and exclusion criteria

All patients where patient contact is within 30 minutes of the injury and who are being transported to the trauma centre by the prehospital clinical team will be considered for inclusion. Exclusion criteria include:

- Patients < 1 year old and > 70 years old.
- Patients with pre-existing neurological disease or neurological trauma.
- Patients with no mechanism for trauma, (e.g. drowning, medical complaints);
- Patients with forehead haematomas or lacerations precluding NIRS probe application;
- Patients where follow-up consent is not later obtained from the patient or family.

Exclusion criteria may be apparent at the accident scene or may be applied upon the collection of additional information that reveals the patient meets exclusion criteria.

### Monitor application

**Figure 1:**
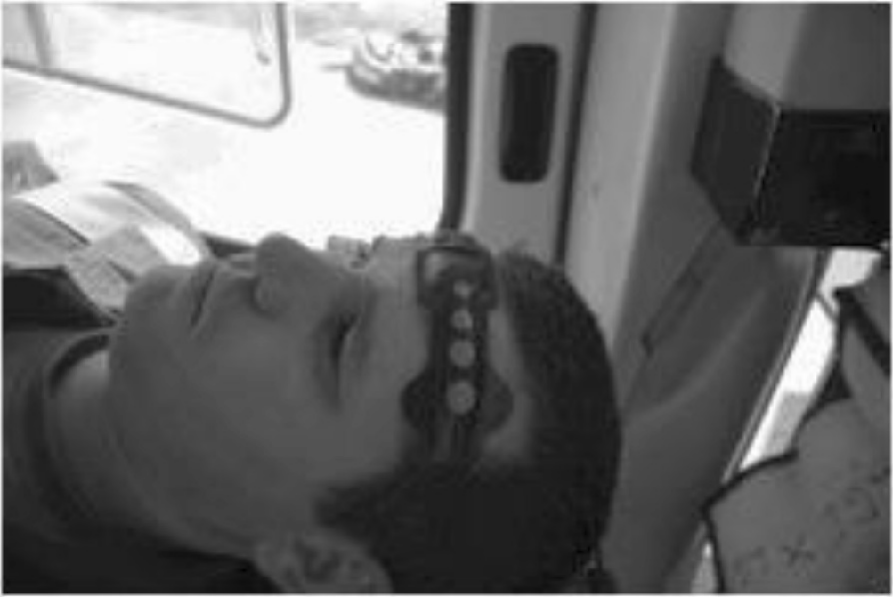
**Cerebral oximetry probes applied to a volunteer during helicopter transport as part of feasibility testing.**

For the purposes of this research, NIRS cerebral oximetry will be applied by a trained researcher as soon as possible after arrival at the patient to ensure the clinical team are able to undertake their standard approach to care. The monitor employed will be a Nonin EQUANOX 7610 Regional Oximetry monitor (TM Nonin Medical, Inc.). This trained observer will apply monitoring probes bifrontally to the forehead of patients and to a peripheral reference site on the forearm (Figure 1). Monitoring will be commenced within a time frame of 30 minutes after the initial injury. The monitoring screen will only show that data capture is proceeding with warning signals if there are issues with sensor contact or signal quality. The EQUANOX 7610 monitor will continuously store the NIRS monitoring data. NIRS monitoring will cease on patient handover at the receiving hospital.

### Data collection

Data collected by the research nurse, who will be blinded to NIRS monitoring information, will include patient demographics already recorded as a routine (available at the time of the injury) and via hospital follow-up. Trauma incident data including incident time and total prehospital duration will also be recorded in initial case documentation.

### NIRS monitoring data

Each NIRS oximetry probe will continually produce 4 streams of data: StO_2_; HbT; HbO_2_; and HbD with values recorded every 4 seconds. Standard clinical monitoring will be recorded at the same time as the NIRS oximetry data captured by the EQUANOX device. This will include peripheral pulse oximetry, end-tidal capnography (where indicated for patient care), heart rate and non-invasive blood pressure and will be updated on the record every ten seconds. This general observations data will be time-matched to the NIRS oximetry monitoring data to allow direct comparisons of values in data analysis, particularly in the signals analysis related to Aim 1. Clinically relevant information such as any degree of prehospital hypoxia or hypotension will therefore be time-matched to the NIRS oximetry monitoring data.

### Patient event and intervention marking

The data capture system for the NIRS oximetry monitor will also facilitate real-time marking of clinical events and interventions by the study observer. This will include real-time marking of bag-mask ventilation, cardiopulmonary resuscitation, anaesthesia with thiopentone, anaesthesia with ketamine, intubation, institution of mechanical ventilation, administration of vasopressor, transfusion, administration of hypertonic saline and seizure activity. Study observers will also have the ability to mark other potential events deemed clinically relevant. This will provide accurate information on interventions undertaken in a manner that is time-matched to the NIRS oximetry monitoring data. Event marking will be combined with NIRS oximetry monitoring data and general observations monitoring to allow reporting of standard interventions and care as previously described [26]. Patient general observations monitoring information and management and intervention details will be presented in conjunction with all outcome data.

### Early phase outcome data

Patient outcome and follow-up data related to initial findings and hospital stay will be obtained from the hospital by the trial research nurse at a time beyond 30 days from the injury and include: (1) Injury Severity Score (ISS) and Abbreviated Injury Scores (AIS) for each body region [27,28]; (2) injuries identified on independent review of first cerebral imaging by computerised tomography (CT) or magnetic resonance imaging (MRI); (3) 24 hour and 30 day mortality; (4) survival to hospital discharge; and, (5) length of intensive care unit (ICU) stay including number of ventilator days.

### Long-term outcomes

Follow-up by the research nurse will be undertaken via phone interview with the patient or appropriately identified relative as previously described [29] to assess and record (1) Glasgow Outcome Scale score (GOS) and Extended Glasgow Outcome Scale (EGOS) score at 6 and 12 months post injury [30]; (2) Disability Rating Scale (DRS) at 6 and 12 months post injury [31] and, (3) Care and Needs Scale (CANS) at 6 and 12 months post injury [32].

### Power analysis

Pre-existing datasets showing the relationship between prehospital NIRS oximetry values and patient injury outcomes in the context of TBI are lacking. To answer Aim 1, a sample size of 242 subjects with severe TBI will provide 90% power to detect correlation of 0.22 or higher [33] in a 2-sided 0.05 level test of the null hypothesis of no correlation between NIRS cerebral oximetry values and admission Glasgow Coma Score (GCS) adjusting for 29 (approximately 12%) unsuccessful NIRS monitoring data collection attempts (25) in a time series based regression analysis. CareFlight’s historical mission database indicates 1210 total recruits will be required to incorporate 242 subjects with severe TBI.

To address Aims 2 and 3, the same number of subjects will be enough to show an incidence of EGOS 4 at six months post-injury of 56% (95%CI 49% to 63%) and demonstrate a clinically important change in EGOS of 1 point associated with NIRS cerebral oximetry value at 80% power to detect a Cochrane-Armitage test for trend in EGOS groups (30% deceased, 20% poor neurological outcome and 50% good neurological outcome) at significance level of 0.05. The expected proportion of EGOS groups have been taken from the subject characteristics from the Head Injury Retrieval Trial [data under analysis] [29].

As for validation of the model for Aims 2 and 3, we will internally validate the models using bootstrapping methods on 1000 samples.

### Subgroups for analysis and reporting

Some patients, particularly those with more severe injuries and those of a different age, are more likely to have monitor findings indicating strong associations with outcomes and are therefore of particular interest. Subgroup analysis will be undertaken on patients with severe TBI (first GCS ≤ 12 and Abbreviated Injury Score (Head) ≥ 3), severe traumatic injury (ISS > 15), and paediatric patients (age < 16 years).

### Data analysis

#### Novel analysis methodology to address aim 1: establishing new analysis methods

A variety of approaches to signals analysis as commonly utilised in biomedical engineering fields will be undertaken. These will include (but not be limited to) time series analysis and pattern recognition and classification undertaken on all monitoring value streams (StO_2_, HbT, HbO_2_ and HbD) for each of the cerebral probes separately, and the peripheral reference probe. These may be matched to ancillary signals including peripheral oxygen saturation and electrocardiograph parameters (RR and QT intervals, RR variability). Comparison may also be made between NIRS oximetry probes. Control group patients with traumatic injuries but no brain injury will be the reference group for analysis of “standard” signals analysis patterns comparisons.

#### Novel analysis methods applied to acute phase outcomes

Analyses undertaken in this way will be tested against the following changes seen on cerebral imaging: no intracranial pathology, intracerebral lesions (haemorrhage or contusion), subdural haematoma, extradural haematoma, traumatic subarachnoid haemorrhage and diffuse injury. Comparison to 24 hour and 30 day mortality will also be undertaken. Control group patients with traumatic injuries but no brain injury will provide data for analysis of “standard” signals analysis patterns.

#### Novel analysis methods applied to long-term outcomes

Analysis as described above will also be assessed against long-term outcomes as grouped by EGOS score at 6 and 12 months as described in the statistical analysis (EGOS < 2, 2–4 and 5–8).

### Outcomes for aim 1

Exploration of a variety of signals analysis approaches to analyse NIRS cerebral oximetry is an explicit component of the study design. Establishment of useful approaches of this nature offer the greatest chance to develop real-time non-invasive monitoring that reflects the underlying pathology that is developing. This has implications not just for future management relevant to all prehospital practitioners but may inform better approaches to utilising NIRS cerebral oximetry in hospital care.

#### Statistical analysis for aim 2: associations of nirs tissue oximetry with acute phase outcomes

The area under the curve for cerebral oximetry value ≤ 45% (minutes x desaturation points ≤ 45%) will be used as an independent variable for statistical analyses as described previously [34]. Patients with one or more episodes of cerebral oximetry value ≤ 45% for 1 minute will be classified as experiencing a decline in cerebral oxygen saturation.

We will define 3 NIRS cerebral oximetry patient groups:

1. NIRS 1 (cerebral oximetry values remain > 45% i.e. area under the curve = 0 min%).
2. NIRS 2 (cerebral oximetry values ≤ 45% for > 0 min up to the median area under the curve for cerebral oximetry value ≤ 45% to be determined).
3. NIRS 3 (cerebral oximetry values ≤ 45% for more than the median area under the curve for cerebral oximetry value ≤ 45% to be determined).

The total duration (minutes) of StO_2_ ≤ 45%, longest duration (minutes)

StO_2_ ≤ 45%, minimum and average StO_2_ will be derived for each patient. These variables will be compared between NIRS 1, NIRS 2 and NIRS 3 groups using Kruskal-Wallis analysis of variance to check the validity of this grouping.

A chi-square test will be used to assess the association between cerebral oximetry values by comparing patients in each of the above groups in their association with the following injury patterns on cerebral imaging: no intracranial pathology, intracerebral lesions present, subdural haematoma, extradural haematoma, traumatic subarachnoid haemorrhage and diffuse injury. The same analysis will also be repeated to establish associations with 24 hour and 30-day mortality and survival to discharge.

In the multivariate analysis, an ordinal logistic regression under a proportional odds model with NIRS 1 as the reference group will be used with the six types of injury on cerebral imaging listed above included as an independent variable after adjusting for ISS, age, sex and other covariates (mean arterial pressure, first recorded GCS, peripheral oximetry, end-tidal CO_2_, HbT) in a stepwise fashion. A score test will be used to verify the proportional odds assumption in the final model. The unadjusted and adjusted odds ratios and 95% confidence intervals will be reported. Significance will be set at P < 0.05.

### Outcomes for aim 2

This analysis will establish the associations between NIRS cerebral oximetry values and types of brain injury. This will underpin new approaches to interpretation of NIRS tissue oximetry values to provide real-time information corresponding with the development of pathology underlying the cerebral oximetry probes, be that swelling and oedema or acute development of blood collections.

#### Statistical analysis for aim 3: associations with long-term outcomes

Utilising the same groupings for NIRS tissue oximetry (NIRS 1 through 3), associations with EGOS (grouped into EGOS < 2, 2–4 and 5–8), GOS and CANS will be tested with the same multivariate analyses and ordinal logistic regression approach described above. This will include adjustment for ISS, age, sex and other covariates. For validation of the model for Aims 2 and 3, we will internally validate the models using bootstrapping methods on 1000 samples. All data analysis will be performed using SPSS version 21 and STATA 13.1 software (StataCorp, College Station, TX).

### Outcomes for aim 3

This analysis will establish simple associations between NIRS cerebral oximetry values and the subsequent long-term functional recovery of patients with TBI. This would potentially allow early identification of patients with more severe injuries to optimise early intervention and engagement of rehabilitation services.

### Research ethics

This project has lead ethics approval from the Sydney Children’s Hospital Network Human Research Ethics Committee (reference 11/SCHN/212). An initial waiver for consent to data collection has been provided as delays to patient care caused by an upfront process would be inappropriate. During the follow-up phase, consent will be obtained from the patient or, where more appropriate, a family member to undertake ongoing data collection and analysis. A Data Safety and Monitoring Committee has also been empanelled. This group will utilise reports from the investigators to confirm that there are no delays or alterations to patient care once the study is under way. Rigorous data security will be maintained for 15 years or, in the case of paediatric patients, until the subject reaches the age of 25.

## Discussion

The ideal prehospital monitoring technology for the patient with TBI would be a non-invasive monitor that provides real-time information regarding conditions in the brain tissue. Appropriately validated this would provide the treating clinician as to what the organ needs at that moment to decrease the size of acute injuries and enhance patient recovery in the long term.

NIRS tissue oximetry may provide such an option. It is easy to apply without delay. In the hospital setting good sensitivity has already been demonstrated for detection of intracranial haematomas preoperatively when correlated with CT or MRI [16-18]. Correlation with invasive monitoring has shown mixed results. Increased length of time with an StO_2_ below 60% has been associated with intracranial hypertension, decreased cerebral perfusion pressure and mortality in a small observational study [33]. In this study, monitoring was applied at the time of cerebral imaging, but this occurred anywhere up to twelve hours after the initial insult.

Research comparing regional oxygen saturation to direct, invasive brain tissue oxygen tension in 22 patients for the 16 hours after severe traumatic brain injury showed moderate accuracy in detecting severe brain hypoxia with area under curve on receiver-operating characteristic curve of 0.82 (with a likelihood ratio of a positive test of 5.3) but a poor ability to detect moderate hypoxia [19]. In this study patients had already passed the phase where they required resuscitation, and had all received neurosurgery. As a result many hours had passed and the subsequent monitoring and analysis is not reflective of early stage pathology.

The prehospital context is precisely about these early stages. Presently available research suggests there is good reason to be interested in cerebral tissue oximetry for this clinical setting, but cannot be considered as knowledge that can be directly transferred. This research will generate new evidence about the potential utility of NIRS tissue oximetry in the prehospital environment. It will be directly related to the prehospital treatment phase with the ability to include recording of general prehospital observations and patient interventions including transfusions and standardised reporting of prehospital airway management [26]. Many of the initial concerns relating to potential interference with cerebral tissue oximetry measurements, including varying venous and arterial compartments in the sampled tissue, oedema, bruising and jaundice have proven to be surmountable with probe placement and looking at monitoring values over time [35]. As obtaining monitoring values has been demonstrated to be feasible in the prehospital setting, there is no reason to believe that this technology cannot be employed in this new clinical context [25].

This study design will facilitate the acquisition of new knowledge directly relevant to prehospital medicine. The data analysis plan will explore associations with broad groupings of regional oxygen saturations as well as more dynamic measures of changing measurements over time through varying approaches to time series analysis. Moreover it will address both early and evolving pathophysiology by exploring associations with findings on cerebral imaging, as well as long-term functional outcomes that may be associated with early cerebral tissue oximetry values. Demonstrating such associations could both deepen our understanding of early pathophysiology of TBI, and provide the basis of future research where clinical interventions are guided by tissue oximetry values.

## Abbreviations

TBI: Traumatic brain injury
NIRS: Near-infrared spectroscopy
HbO_2_: Oxyhaemoglobin
HbD: Deoxyhaemoglobin
HbT: Total tissue haemoglobin index
StO_2_: Regional oxygen saturation
ISS: Injury severity score
AIS: Abbreviated injury score
CT: Computerised tomography
MRI: Magnetic resonance imaging
ICU: Intensive Care Unit
GOS: Glasgow outcome score
EGOS: Extended Glasgow outcome score
DRS: Disability rating scale
CANS: Care and needs scale
GCS: Glasgow come score
CO_2_: Carbon dioxide
